# Immunotherapy in Pancreatic Cancer: Why Do We Keep Failing? A Focus on Tumor Immune Microenvironment, Predictive Biomarkers and Treatment Outcomes

**DOI:** 10.3390/cancers14102429

**Published:** 2022-05-14

**Authors:** Alessandro Di Federico, Mirta Mosca, Rachele Pagani, Riccardo Carloni, Giorgio Frega, Andrea De Giglio, Alessandro Rizzo, Dalia Ricci, Simona Tavolari, Mariacristina Di Marco, Andrea Palloni, Giovanni Brandi

**Affiliations:** 1 Division of Medical Oncology, IRCCS Azienda Ospedaliero-Universitaria di Bologna, Via Albertoni, 15, 40138 Bologna, Italy; mirta.mosca@studio.unibo.it (M.M.); rachele.pagani@studio.unibo.it (R.P.); riccardo.carloni2@studio.unibo.it (R.C.); andrea.degiglio2@unibo.it (A.D.G.); mariacristina.dimarco@unibo.it (M.D.M.); andrea.palloni@aosp.bo.it (A.P.); giovanni.brandi@unibo.it (G.B.); 2 Department of Specialized, Experimental and Diagnostic Medicine, University of Bologna, Via Giuseppe Massarenti, 9, 40138 Bologna, Italy; simona.tavolari@unibo.it; 3 Osteoncology, Bone and Soft Tissue Sarcomas, and Innovative Therapies, IRCCS Istituto Ortopedico Rizzoli, 40136 Bologna, Italy; giorgio.frega2@unibo.it; 4 Struttura Semplice Dipartimentale di Oncologia Medica per la Presa in Carico Globale del Paziente Oncologico “Don Tonino Bello”, I.R.C.C.S. Istituto Tumori “Giovanni Paolo II”, Viale Orazio Flacco 65, 70124 Bari, Italy; a.rizzo@oncologico.bari.it; 5 Departmental Unit of Medical Oncology, ASL BA, 20142 Milan, Italy; dalia.ricci@asl.bari.it

**Keywords:** pancreatic cancer, immunotherapy, tumor microenvironment, immune biomarkers, PD-L1, TMB

## Abstract

**Simple Summary:**

In pancreatic cancer, immunotherapy and targeted therapies have not brought about the therapeutic revolution that has been observed in other malignancies. Among the reasons to explain this difference is the possibly crucial role played by the pancreatic tumor microenvironment, which has unique features and is different from that of other neoplasms. The aim of this review is to provide a comprehensive overview of the distinctive tumor immune microenvironment of pancreatic cancer and to summarize existing data about the use of immunotherapy and immune biomarkers in this cancer.

**Abstract:**

The advent of immunotherapy and targeted therapies has dramatically changed the outcomes of patients affected by many malignancies. Pancreatic cancer (PC) remains one the few tumors that is not treated with new generation therapies, as chemotherapy still represents the only effective therapeutic strategy in advanced-stage disease. Agents aiming to reactivate the host immune system against cancer cells, such as those targeting immune checkpoints, failed to demonstrate significant activity, despite the success of these treatments in other tumors. In many cases, the proportion of patients who derived benefits in early-phase trials was too small and unpredictable to justify larger studies. The population of PC patients with high microsatellite instability/mismatch repair deficiency is currently the only population that may benefit from immunotherapy; nevertheless, the prevalence of these alterations is too low to determine a real change in the treatment scenario of this tumor. The reasons for the unsuccess of immunotherapy may lie in the extremely peculiar tumor microenvironment, including distinctive immune composition and cross talk between different cells. These unique features may also explain why the biomarkers commonly used to predict immunotherapy efficacy in other tumors seem to be useless in PC. In the current paper, we provide a comprehensive and up-to-date review of immunotherapy in PC, from the analysis of the tumor immune microenvironment to immune biomarkers and treatment outcomes, with the aim to highlight that simply transferring the knowledge acquired on immunotherapy in other tumors might not be a successful strategy in patients affected by PC.

## 1. Introduction

Pancreatic cancer (PC) is one of the leading causes of cancer death worldwide [1]. Despite the incidence of PC being much lower than that of other malignancies, such as lung, breast, colorectal, and prostate tumors, it was the third highest cause of cancer-related death in 2021 and is projected to rise to the second position by 2030 [1,2]. As much as 52% of pancreatic tumors show distant dissemination at diagnosis, which confers a 5-year survival rate of only 3%, highlighting the remarkable need to develop more effective therapeutic strategies [1]. Currently, chemotherapy represents the mainstay of the treatment of patients affected by advanced PC [3]. Despite the success of immunotherapy and targeted therapies in many solid tumors in the last decade, these agents were not shown to provide a significant benefit to PC patients [4]. The peculiar pancreatic tumor microenvironment (TME) certainly plays an important role in the lack of success of most therapeutic strategies, including immunotherapy. Studying the tumor immune microenvironment (TIME) is therefore essential to understanding the complex interactions between all the actors involved in cancer immune escape mechanisms and treatment resistance and to learning how to exploit the immune system against PC cells. Moreover, the identification of key features that are able to predict the outcomes of immune-based strategies is necessary to identify that a small proportion of patients that would benefit from these types of treatments. In the current paper, we provide a comprehensive review of the literature, focusing on the immune contexture of PC and its impact on therapeutic strategies aiming to reactivate the immune system against cancer cells. We also analyzed the current knowledge regarding potential predictive and prognostic immune biomarkers and provide an up-to-date overview of immunotherapy in this aggressive tumor.

## 2. The Pancreatic Tumor Immune Microenvironment

**Figure 1:**
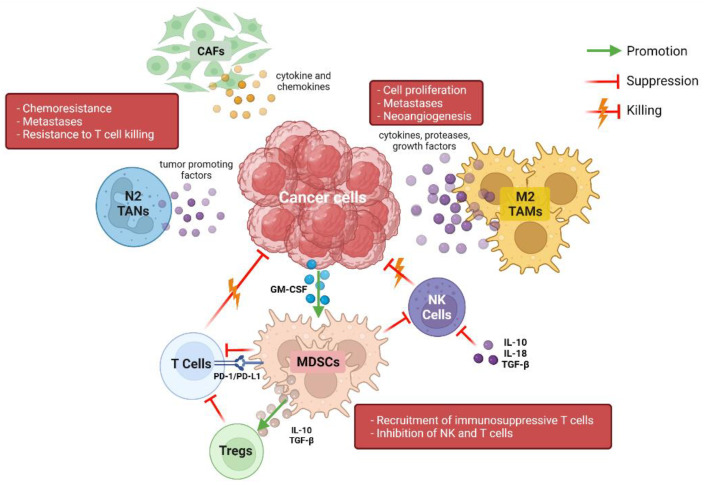
Representation of immune cross talk in the complex pancreatic tumor microenvironment. CAFs: cancer-associated fibroblasts; TANs: tumor-associated neutrophils; TAMs: tumor-associated macrophages; NK: natural killer; MDSC: myeloid-derived suppressor cells; PD-1: programmed death-1; PD-L1: programmed death-ligand 1; GM-CSF: granulocyte-macrophage colony-stimulating factor. Created with BioRender (www.biorender.com (accessed on 3 May 2022)).

Pancreatic ductal adenocarcinoma (PDAC) is characterized by a dense and rich stroma, composed of immune cells, blood vessels, fibroblasts, and many other types of cells [5]. Interactions between stroma and cancer cells are responsible for tumor growth, proliferation, and survival, as well as for drug responsiveness and resistance; therefore, the analysis and understanding of TME and TIME are essential to developing valid therapeutic strategies. Many studies have been conducted to understand the association between TIME composition and patient prognoses, but reliable data are still lacking. Different types and subtypes of immune cells are present in TIME in varying percentages and can interact with each other in multiple ways, determining a multitude of different effects (Figure 1) [6,7]. Myeloid cells represent a major component of stroma cells and the high number of tumor-associated macrophages (TAMs) seems to inversely correlate with prognosis in PDAC patients [8,9]. TAMs can promote neo-angiogenesis, cancer cell proliferation, and metastases through the release of cytokines, proteases, and growth factors, such as vascular endothelial growth factor (VEGF) [10,11]. Moreover, TAMs are able to influence the activity of cytidine deaminase, responsible for gemcitabine metabolization, conferring resistance to this drug [12]. In TME, TAMs are differentiated into two subpopulations called “M1” and “M2”, which have opposite roles: M2 have mainly anti-inflammatory functions, while M1 exert anti-cancer effects through the release of pro-inflammatory cytokines [13,14]. Myeloid-derived suppressive cells (MDSCs), which are recruited in PDAC stroma by cancer cells through the production of the granulocyte-macrophage colony-stimulating factor (GM-CSF), play an important anti-inflammatory function in PDAC TIME [15,16]. MDSCs inhibit both innate and adaptive immune responses; in fact, they can block natural killer cells (NK cells) with a direct-contact mechanism and are able to upregulate the expression of programmed death-1 (PD-1) on their surface, favoring the suppression of T-cell activation [17,18,19]. Moreover, MDSCs produce interleukin-10 (IL-10) and transforming growth factor-β (TGF-β), which recruit immunosuppressive regulatory T cells (Tregs) [20]. NK cells are altered in several ways in pancreatic TIME; in normal conditions, NK cells exert direct killing functions on tumor cells, which are independent from antigen stimulation and mediated by cell receptors, such as CD16, natural killer group 2 membrane D (NKG2D), DNAM-1, and natural cytotoxicity receptors (NCRs). Instead, in PDAC, they seem to be polarized towards a less-functioning and tumor-promoting phenotype, a result of a series of complex interactions between NK cells, cancer cells, and other immune cells [21,22]. The increased production of interleukin-10, interleukin-18, and TGFβ and the downregulation of activating receptors are some of the causes of the reduced NK cell function seen in PDAC [23,24]. These “polarized” NK cells are characterized by a lower production of cytotoxic granzyme B and perforin and lower expression of the chemokine receptor CXCR2, with a subsequent impaired tumor trafficking and reactivity [25,26].

Neutrophils have a controversial role in PDAC development. In TIME, tumor-associated neutrophils (TANs) are polarized in two subpopulations named “N1” and “N2” by TGFβ and IFNα, respectively [27]. N1 neutrophils have pro-inflammatory effects and stimulate the recruitment and activation of CD8+ T cells [28], while N2 neutrophils release tumor-promoting factors such as metalloproteinase (MMPs), neutrophil elastase (NE), reactive oxygen, and nitrogen species [29,30]. IL-17, mainly produced by Tregs, acts as an indirect inducer factor of neutrophil extracellular traps production (NETs) [31] and NET formation can promote liver metastasis and immune checkpoint inhibitor (ICI) resistance by blocking CD8+ T cells. Neutrophils are also responsible for the production of lipocalin-2, an adipokine that is implicated in stromal remodeling and tumor cell activation [32].

Tregs have several anti-inflammatory effects, which are primarily mediated by their ability to suppress T-cell activity and are present from the initial stages of the tumorigenesis process [33]. In fact, the number of Treg and Th-17 cells is elevated in premalignant lesions, such as intraductal papillary mucinous neoplasm (IPMNs) and pancreatic intraepithelial neoplasia (PanIN), and seems to be directly correlated with tumor stage and poor prognosis [34,35,36].

In pancreatic TIME, an increased Th2/Th1 tumor-infiltrating lymphocytes ratio has been largely documented [37,38,39]. Th2 role is mediated by the master transcription factor GATA3, which induces M2 macrophage activation and stimulate cancer cell proliferation. Tumor-promoting function is also directly explained by the increased activation of STAT3, AKT, and MAPK pathways [40]. Th2 enrichment in TIME is mediated by different stimulating agents produced by dendritic cells, B cells, TAMs, microbiota, and cancer-associated fibroblast (CAFs) [41,42,43].

Among T cells, CD8+ T lymphocytes have a direct and cytotoxic effect on cancer cells. However, tumors can induce CD8+ T exhaustion, a state characterized by shorter cell survival and impaired effector abilities. A recent study showed that cytotoxic T cells are associated with a longer survival only in patients whose tumors overexpressed targets for pyroptosis and ferroptosis, which are two mechanisms of tumor cell killing. The same study also noted that a high number of T cells overexpressing ribosome-related proteins correlates with better outcomes [44].

As previously mentioned, non-immune cells, such as stellate cells and CAFs, are present in PDAC TIME and play essential roles [45,46,47]. CAFs can be divided into three specific subpopulations. The first is located near cancer cells and has myofibroblastic and anti-tumor features, while the second one is activated by IL-1 and contributes to the generation of an immunosuppressed TIME [48,49]. The role of the third subpopulation, named antigen-presenting CAFs, has not been well established, but they seem to have an immune-suppressive role [47]. Interactions between CAFs, cancer cells, and immune cells are variable and complex. Intra-tumoral or gut commensal bacteria may indirectly increase the expression of IL-1 and consequently the activation of the second subpopulation of CAFs, which is responsible for the production of IL-6, IL-33, CXCL12, IL-8, and other molecules. These cytokines and chemokines promote tumor angiogenesis and bestow chemoresistance and resistance to T-cell killing activity [50,51,52]. Myofibroblastic CAFs are stimulated by TGFβ, which suppresses T-cell activity, polarizes macrophages to M2 subtypes, and is implicated in cell growth, epithelial-to-mesenchymal transition, and extracellular matrix production [53,54]. These functions have made TGFβ an attractive target in pancreatic cancer treatment and have led to the evaluation of its inhibitor, Galunisertib, in combination with chemotherapy in a first-line setting [55]. Other studies have been conducted using pegvorhyaluronidase alfa (PEGPH20), a molecule which degrades hyaluronic acid in TME, with contrasting results [56,57].

Finally, the contribution of microbiota in the generation of an immune-suppressed TIME in PDAC cannot be overlooked. In murine models, bacterial ablation resulted in a lower presence of M2 macrophages and a major presence of CD8+ T-cells and PD-1 expression in TME [58]. ICIs and antibiotics could therefore have a synergistic role, but the toxicities and adverse events of this combination strategy should be further studied and carefully considered.

## 3. Prognostic and Predictive Immune Biomarkers

The identification of reliable biomarkers able to predict the outcomes of PDAC patients treated with immunotherapy is of paramount importance. In fact, despite the disappointing results generally obtained with ICIs, a small proportion of patients seem to derive durable benefit from immunotherapy [4]. The peculiar immune features of the TIME may predict the prognosis and immune susceptibility of PC patients. The presence of a high tumor mutational burden (TMB) is correlated with a high tumor neoantigen load and is considered as a gross indicator of enhanced immunotherapy efficacy [59]. However, different thresholds have been adopted to define a TMB as “high”, varying across studies and tumor type [60,61]. Some authors have proposed that a high TMB should correspond to the highest TMB quintile in each histology [61]. Median TMB is typically very low in PDAC, ranging around 1–4 mutations/Mb in pancreatic ductal adenocarcinoma and, according to a recent systematic review of the literature, the proportion of TMB-high PDAC is only 1.1% [62,63,64]. Most studies adopted the cut-off of ≥20 mutations/Mb to define a TMB-high PDAC. Interestingly, these tumors show a higher prevalence of mucinous-colloid and medullary histology, which are both usually very rare (<2%), and have a distinctive genomic landscape, including mutations in BRAF, ERBB2, BRCA2, and POLE genes and a high presence (approximately 60%) of high microsatellite instability or mismatch repair deficiency (MSI-H/dMMR) [65]. Among eight patients affected by pancreatic ductal adenocarcinoma with high-TMB and treated with programmed death-1 (PD-1) inhibitors, one patient had stable disease (SD), five had partial responses (PR), and two patients had complete responses (CR), both with MSI-H/dMMR [65]. These data support further studies on the role of TMB as a potential biomarker for PDAC patients treated with immunotherapy.

Programmed death-ligand 1 (PD-L1) expression represents a predictive biomarker of response to ICI in many tumors. In PDAC, a positive PD-L1 expression can be found in approximately 30–40% of cases and is correlated with low tumor-infiltrating lymphocytes, particularly CD8+ cells, and a poor prognosis [66,67]. A recent study identified four PC patterns based on PD-L1 expression on tumor cells (TC) and immune cells (IC): “adaptive-1” (TC: 0, IC > 1%), “adaptive-2” (TC > 1% to <25%, IC > 1%), “constitutive” (TC ≥ 25%, IC: 0), and “combined” (TC ≥ 25%, IC > 1%) [66]. “Adaptive-1” tumors showed a T cell inflamed TIME, characterized by high CD3+, CD4+ and CD8+ cells and PD1+ T cells and low CD68+ macrophages, including the M2-polarized subpopulation, and were associated with the longest survival. Conversely, “constitutive” tumors had reduced IC, except for CD68+ TAMs, and had worst outcomes [66]. A recent meta-analysis exploring the prognostic role of immune infiltration in PDAC documented a negative prognostic impact of CD163+ M2 polarized macrophages [67]. Instead, a high infiltration of CD4+ and CD8+ lymphocytes was associated with longer disease-free survival [67]. PD-L1 expression has been shown to correlate with Cancer-Forkhead box P3, which can promote immune evasion in PDAC by recruiting Forkhead box P3+ Treg cells via CCL5 upregulation [68]. Consistently, ICIs seem to enhance the anti-tumor efficacy of the CCL5 blockade [68]. PD-L1 expression appears to also be positively influenced by mutations in RAS, MYC, and MLL1 genes [69,70,71]. Despite the preclinical rationale, data from clinical studies correlating PD-L1 expression with tumor response to ICI have been inconsistent. The presence of MSI-H/dMMR currently represents the only reliable predictive biomarker of response to ICI in PDAC [72]. Unfortunately, MSI-H/dMMR PDAC represent a very small population, with a prevalence of less than 3% [73,74]. Since this population partially overlaps with TMB-high PDAC, MSI-H/dMMR PDAC share with them several characteristics, such as a higher prevalence of medullary and mucinous/colloid histology and a peculiar genomic background, with more frequent JAK mutations and significantly less common KRAS and TP53 mutations compared to microsatellite stable PDAC [74]. In addition to its recognized oncogenic role, mutant KRAS seems to drive the formation of immunosuppressed TIME, directly preventing innate and adaptative anti-tumor immunity by modulating the levels of cell surface HLA class I and by regulating the expression of CD47 and PD-L1 [75,76,77]. Moreover, KRAS mutations induce a desmoplastic TME composed of suppressive immune cells [76]. Therefore, KRAS inhibition with novel therapies could be a new approach to render PDAC sensitive to immunotherapy [78].

### Immunotherapy: Searching for the Right Key

Finding a valid strategy to exploit the host immune system against pancreatic cancer cells is challenging. Antibodies targeting CTLA-4, PD-1, and PD-L1 immune checkpoints demonstrated their efficacy in many tumors, including malignant melanoma, lung, urothelial, and renal cancers. However, the results obtained in patients affected by PDAC have been largely disappointing [4,79]. The number of cells expressing PD-1 and PD-L1 is lower in PDAC compared with tumors where immunotherapy demonstrated an established efficacy, such as malignant melanoma [80]. However, several other immune inhibitory molecules are frequently upregulated, including LAG-3, galectins, TIGIT, and V-domain Ig suppressor of T-cell activation (VISTA) [81,82,83,84]. Efforts to enhance immune infiltration in PDAC TME included targeting CXCR4, which can be inhibited to increase T-cell chemotaxis. In fact, the combination of PD-1 and CXCR4 inhibition resulted in enhanced T-cell expansion and tumor cell death in preclinical models [85]. CD40 activation may represent a strategy to reverse T-cell exhaustion, enhancing the anti-cancer effects of the TIME. Consistently, agonistic CD40 antibodies were shown to increase T-cell mediated cancer death and, in combination with chemotherapy, may rescue ICI sensitivity [86,87,88,89]. Recently, a phase I trial documented the safety profile and the potential activity of combining sotigalimab, a CD40 agonistic monoclonal antibody, with chemotherapy, with or without the PD-1 inhibitor nivolumab, in previously untreated metastatic PDAC [89]. Among 24 dose-limiting toxicity-evaluable patients, a 58% objective response rate (ORR) was reached, and there were no safety concerns. Many clinical trials tested ICI as a single-agent or combined with other agents that may enhance immunotherapy efficacy. CTLA-4 inhibition proved to be poorly effective, either alone or combined with PD-L1 inhibition in two phase II trials testing, respectively, single-agent ipilimumab and durvalumab, with or without tremelimumab, in patients with advanced PDAC [90,91]. Similarly, the addition of single-agent ICI or a dual immune blockade to standard chemotherapy with gemcitabine and nab-paclitaxel did not lead to significant activity or survival improvements [92,93]. In the neoadjuvant setting, adding pembrolizumab to chemoradiation therapy did not improve the efficacy of chemoradiation alone and did not result in significant changes to the infiltration of several immune cell subsets in the TIME [94]. An interesting phase II pilot trial tested the addition of nivolumab and paricalcitol to triple-agent chemotherapy, represented by a combination of nab-paclitaxel, cisplatin, and gemcitabine, in 10 patients with previously untreated metastatic PDAC [95]. Despite the small sample size, the encouraging objective response rate of 80% and disease control rate of 100% warranted further investigation.

Combining ICI with vaccines represents another viable strategy to enhance immunotherapy efficacy. The combination of ipilimumab and GVAX, a granulocyte-macrophage colony-stimulating factor (GM-CSF) cell-based vaccine, enhanced the T-cell repertoire and led to a numerically better overall survival (OS) compared to single-agent ipilimumab (5.7 months vs 3.6 months, p = 0.072), although not reaching statistical significance [96]. Recently, the results of a trial testing the combination of GVAX alone, combined with nivolumab or with nivolumab plus the anti-CD137 urelumab as neoadjuvant or adjuvant therapy for patients with resectable PDAC were presented at the 2022 ASCO Gastrointestinal Cancers Symposium [97]. The full combination regimen (GVAX plus nivolumab plus urelumab) was associated with an improved pathologic response, numerically longer disease-free survival and OS, not reaching statistical significance, and mild toxicity. A phase III trial testing algenpantucel-L, an allogenic vaccine made up of αGal-expressing engineered PDAC cell lines, in combination with adjuvant chemotherapy and chemoradiotherapy did not demonstrate significant efficacy improvements, despite the promising results in terms of disease-free survival (DFS) and OS reported by the phase II single-arm trial [98,99].

Among vaccination strategies, oncolytic viruses have been largely explored in PC, alone or in combination with conventional therapies. The term “oncolytic viruses” refers to natural or genetically modified viruses used as therapeutic agents in various malignancies. These viruses can act directly, causing cancer cell lysis, and also indirectly, modifying TME and promoting cancer regression [100,101,102]. Few encouraging results have been obtained in preclinical and clinical studies evaluating the role of vaccinia, reovirus, herpes simplex-1, and adenovirus as potential oncolytic viruses, probably because of the density and subsequent low penetrability of pancreatic cancer TME that limits the access of viruses [103].

Pelareorep, an isolate of a strain of reovirus, has been studied in combination with gemcitabine in a phase II trial, showing high viral replication in tumor cells and good tolerance [104]. It has been also studied in combination with pembrolizumab and chemotherapy in pretreated patients in a recent phase Ib trial, showing promising results and confirming its safety [105].

Bruton tyrosine kinase (BTK) is expressed by several immune cells, including macrophages [106]. In murine models of PDAC, BTK inhibition promoted the conversion from an M2-like to M1-like macrophage and the differentiation of CD8 T-cells and was associated with enhanced tumor shrinkage when combined with gemcitabine [43]. Acalabrutinib, a BTK inhibitor, was investigated, with or without the addition of pembrolizumab, in patients with previously treated advanced PDAC in a phase II randomized trial [107]. In the combination arm, PR and SD were 7.9% and 21.1%, respectively, vs. 0% and 14.3% in the acalabrutinib alone arm. The presence of high microsatellite instability (MSI-H) was found in approximately 1–2% of PDAC [74,108]. Similarly, a high tumor mutational burden (TMB), defined as ≥20 mutations/megabase, is extremely rare in PDAC [109]. The KEYNOTE-158 trial evaluated the efficacy of pembrolizumab in patients with MSI-H/mismatch repair-deficient advanced non-colorectal cancers [60]. Among 22 MSI-H PDAC patients who received the PD-1 inhibitor, ORR was observed in four (18.2%), including one CR, with a satisfactory median duration of response of 13.4 months. However, the overall results were poor compared to those of patients harboring other tumors, such as gastric and endometrial cancer or cholangiocarcinoma, either in terms of ORR or median PFS (2.1 months) and OS (4.0 months). Currently ongoing phase II and III trials testing immune-based strategies in patients affected by PC are summarized in Table 1.

## 4. Conclusions

Immunotherapy and targeted therapy did not yield practice-changing results in pancreatic cancer, probably because PDAC TME and TIME are peculiar compared to most tumors, as is the genomic landscape that accompanies this disease. The simple transfer of knowledge about the efficacy of immunotherapy and targeted therapy in other neoplasms is not sufficient to obtain promising results in pancreatic cancer and needs to be integrated with a deeper comprehension of TME cells and their interactions. More research efforts are crucial to better select patients that could benefit from immunotherapy and to develop efficient TME modification mechanisms that could make the tumor more immunosensitive. Identifying biomarkers able to predict clinical outcomes of pancreatic cancer patients treated with immunotherapy and targeted therapies, regardless of the disease setting, is of paramount importance.

## Figures and Tables

**Table 1:** Ongoing phase II–III clinical trials evaluating the use of immune-checkpoint inhibitors selectively in patients with pancreatic cancer.

NCT (Acronym)	Phase	Number of Patients	Setting	Treatment Arms	Primary Endpoint	Status
NCT03989310	I/II	50	Locally advanced/Metastatic	(1) Manganese chloride + Nab-paclitaxel + Gemcitabine + Anti-PD-1 antibody (2) Nab-paclitaxel + Gemcitabine + Anti-PD-1 antibody	Safety DCR	Recruiting
NCT04548752	II	88	Maintenance, BRCA mutated	(1) Olaparib + Pembrolizumab 29 Olaparib	PFS	Recruiting
NCT04156087	II	20	Locally Advanced	MIS-MWA + Durvalumab + Tremelimumab	PFS	Recruiting
NCT05116917	II	30	Metastatic	Nivolumab + Ipilimumab + Influenza vaccine + SBRT	ORR	Recruiting
NCT03161379	II	30	Borderline resectable, neoadjuvant	Cyclophosphamide + Nivolumab + GVAX + SBRT	CD8 count (cells/mm^3^) in the tumor microenvironment	Active, not recruiting
NCT04324307	I/II	60	Locally Advanced/ Metastatic	(1) 2nd line PD-L1/CTLA4 inhibitor (2) 1st line PD-L1/CTLA4 inhibitor + gemcitabine/nab-paclitaxel (3) 1st line PD-L1/CTLA4 inhibitor + FOLFIRINOX	ORR	Recruiting
NCT03193190	Ib/II	290	Metastatic	Severals, combinations of Nab-Paclitaxel, Gemcitabine, Oxaliplatin, Fluorouracil, Atezolizumab, Cobimetinib, PEGPH20, BL-8040, Selicrelumab, Bevacizumab, RO6874281, AB928, Tiragolumab and Tocilizumab.	ORR Safety	Active, not recruiting
NCT04361162	II	30	Metastatic	Nivolumab + Ipilimumab + Radiation	ORR	Recruiting
NCT04543071	II	10	Metastatic	Motixafortide, Cemiplimab, Gemcitabine, Nab-Paclitaxel	ORR	Recruiting
NCT03336216	II	179	Advanced, pretreated	(1) Gemcitabine/Nab-Paclitaxel or 5-FU/Leucovorin/Irinotecan Liposome (2) Cabiralizumab + Nivolumab (3) Gemcitabine + Nab-Paclitaxel + Cabiralizumab + Nivolumab (4) Cabiralizumab + Nivolumab + FOLFOX	PFS	Active, not recruiting
NCT03977272	III	110	Metastatic	(1) modified-FOLFIRINOX/FOLFIRINOX (2) modified-FOLFIRINOX/FOLFIRINOX + anti PD-1 antibody 200 mg	OS	Recruiting
NCT03983057	III	830	Locally advanced/borderline resectable	(1) modified-FOLFIRINOX (2) modified-FOLFIRINOX + anti PD-1 antibody 3 mg/kg	PFS	Recruiting
NCT04377048	II	38	Metastatic	Tegafur-Gimeracil-Oteracil + Nivolumab + Gemcitabine	ORR	Not yet recruiting
NCT04493060	II	20	Metastatic, germline or somatic BRCA1/2 and PALB2 related cancer	Dostarlimab + Niraparib	DCR	Recruiting
NCT02648282	II	58	Locally advanced	Cyclophosphamide + Pembrolizumab + GVAX + SBRT	DMFS	Active, not recruiting
NCT04887805	II	28	Maintenance after 1st or 2nd line chemotherapy	Lenvatinib + Pembrolizumab	PFS	Recruiting
NCT04247165	I/II	20	Locally advanced	Nivolumab + Ipilimumab + Gemcitabine + Nab-paclitaxel + SBRT	Safety	Recruiting
NCT05093231	II	20	Metastatic	Pembrolizumab + Olaparib	ORR	Recruiting
NCT04940286	II	30	Resectable/borderline resectable, neoadjuvant	Durvalumab + Oleclumab + Nab-paclitaxel + Gemcitabine	Major pathological response rate (≤5% viable tumor cells) Safety	Recruiting
NCT02305186	I/II	68	Resectable/borderline resectable, neoadjuvant	(1) chemoradioterapy (with Capecitabine) (2) chemoradioterapy (with Capecitabine) + Pembrolizumab	TILs per HPF Safety	Recruiting
NCT04827953	I/II	24	Advanced	Gemcitabine + nab-paclitaxel + NLM-001 + Zalifrelimab	ORR	Recruiting
NCT03563248	II	160	Resectable/borderline resectable/locally advanced, neoadjuvant	(1) FOLFIRINOX → SBRT → Surgery (2) FOLFIRINOX + Losartan → SBRT + Losartan → Surgery (3) FOLFIRINOX + Losartan → SBRT + Nivolumab + Losartan → Surgery (4) FOLFIRINOX × 8 → SBRT + Nivolumab → Surgery	Proportion of patients with R0 resection	Recruiting
NCT04177810	II	21	Metastatic	Plerixafor + Cemiplimab	ORR	Recruiting
NCT05014776	II	20	Metastatic, pretreated	Tadalafil + Pembrolizumab + Ipilimumab + CRS-207	irORR	Recruiting
NCT03190265	II	63	Metastatic, pretreated	(1) Cyclophosphamide + Nivolumab + Ipilimumab + GVAX + CRS-207 (2) Nivolumab + Ipilimumab + CRS-207	ORR	Active, not recruiting
NCT02907099	IIb	18	Metastatic	CXCR4 antagonist BL-8040 + Pembrolizumab	ORR	Active, not recruiting
NCT04116073	II	25	Unresectable/Metastatic, pretreated	INCMGA00012 (PD-1 antibody)	DCR4	Recruiting
NCT04753879	II	38	Metastatic	Nab-paclitaxel + Gemcitabine + Cisplatin + Irinotecan + Capecitabine → Maintenance with Pembrolizumab + Olaparib	PFS	Recruiting
NCT03767582	I/II	30	Locally advanced	(1) SBRT + Nivolumab + CCR2/CCR5 dual antagonist (2) SBRT + Nivolumab + GVAX + CCR2/CCR5 dual antagonist	Safety Immune response	Recruiting
NCT03727880	II	36	Neoadjuvant/Adjuvant, resectable at diagnosis	(1) Pembrolizumab + Defactinib (2) Pembrolizumab	pCR	Recruiting
NCT04624217	Ib/II	54	Advanced, pretreated	Gemcitabine + Nab-paclitaxel + SHR-1701	ORR RP2D	Active, not recruiting

PD-1: programmed death-1; DCR: disease control rate; PD-L1: programmed death-ligand 1; PFS: progression-free survival; MIS-MWA: Minimally Invasive Surgical Microwave Ablation; SBRT: Stereotactic Body Radiation Therapy; GVAX: granulocyte-macrophage colony-stimulating factor (GM-CSF) gene-transfected tumor cell vaccine; CTLA-4: Cytotoxic T-Lymphocyte Antigen 4; OS: overall survival; DCR: disease control rate; DMFS: distant metastasis-free survival; TILs: tumor-infiltrating Lymphocytes; HPF: high-powered field; irORR: Objective response rate using immune Response Evaluation Criteria for Solid Tumors (iRECIST); CXCR4: C-X-C Motif Chemokine Receptor 4; DCR4: disease control rate at 4 months; CCR2: Chemokine (C-C motif) receptors 2; CCR5: Chemokine (C-C motif) receptors 5; pCR: pathologic complete response; RP2D: recommended phase 2 dose.
